# Non-invasive focus localization, right ventricular epicardial potential mapping in patients with an MRI-conditional pacemaker system ‐ a pilot study

**DOI:** 10.1007/s10840-015-0054-9

**Published:** 2015-09-14

**Authors:** A W Maurits van der Graaf, Pranav Bhagirath, Jacques de Hooge, Hemanth Ramanna, Vincent J H M van Driel, Natasja M S de Groot, Marco J W Götte

**Affiliations:** Department of Cardiology, Haga Teaching Hospital, Leyweg 275, 2545 CH The Hague, The Netherlands; Department of Cardiology, Erasmus Medical Center, Rotterdam, The Netherlands

**Keywords:** Non-invasive imaging, Inverse potential mapping, Body surface potential mapping, Computational cardiac electrophysiology, MRI-conditional pacemaker systems

## Abstract

**Background:**

With the advent of magnetic resonance imaging (MRI) conditional pacemaker systems, the possibility of performing MRI in pacemaker patients has been introduced. Besides for the detailed evaluation of atrial and ventricular volumes and function, MRI can be used in combination with body surface potential mapping (BSPM) in a non-invasive inverse potential mapping (IPM) strategy. In non-invasive IPM, epicardial potentials are reconstructed from recorded body surface potentials (BSP). In order to investigate whether an IPM method with a limited number of electrodes could be used for the purpose of non-invasive focus localization, it was applied in patients with implanted pacing devices. Ventricular paced beats were used to simulate ventricular ectopic foci.

**Methods:**

Ten patients with an MRI-conditional pacemaker system and a structurally normal heart were studied. Patient-specific 3D thorax volume models were reconstructed from the MRI images. BSP were recorded during ventricular pacing. Epicardial potentials were inversely calculated from the BSP. The site of epicardial breakthrough was compared to the position of the ventricular lead tip on MRI and the distance between these points was determined.

**Results:**

For all patients, the site of earliest epicardial depolarization could be identified. When the tip of the pacing lead was implanted in vicinity to the epicardium, i.e. right ventricular (RV) apex or RV outflow tract, the distance between lead tip position and epicardial breakthrough was 6.0 ± 1.9 mm.

**Conclusions:**

In conclusion, the combined MRI and IPM method is clinically applicable and can identify sites of earliest depolarization with a clinically useful accuracy.

**Electronic supplementary material:**

The online version of this article (doi:10.1007/s10840-015-0054-9) contains supplementary material, which is available to authorized users.

## Introduction

Invasive electrophysiological procedures are often complicated by considerable fluoroscopic time, non-inducibility of the arrhythmia or hemodynamic instability of the patient [1]. Therefore, pre-procedural and non-invasive localization of arrhythmogenic foci may improve the clinical outcome and reduce the duration of the invasive procedures [2].

To this intent, over the last decade various non-invasive mapping strategies, e.g. electrocardiographic imaging (ECGI) [3], non-invasive imaging of cardiac electrophysiology (NICE) [4] and AMICARD [5] have been introduced. Despite numerous reports on the possible advantages of non-invasive mapping, this approach has not yet advanced into daily clinical practice as a routine tool. This is either due to the impracticability associated with the use of up to 254 torso electrodes or to the limited data available from *in vivo* studies with respect to the accuracy and validity of the estimated epicardial potentials or intramural activation times.

With the advent of magnetic resonance imaging (MRI) conditional pacemaker systems, the possibility of performing MRI in pacemaker patients has been introduced [6]. Besides for the detailed evaluation of atrial and ventricular volumes and function, MRI can be used in combination with body surface potential mapping (BSPM) in a non-invasive inverse potential mapping (IPM) strategy.

This pilot study investigated the feasibility of non-invasive focus localization using a limited number of 62 electrodes in patients with an implanted MRI-conditional pacemaker system. Ventricular paced beats were used to simulate ectopic foci. The estimated site of earliest epicardial breakthrough was compared to the position of the ventricular lead tip, and the distance between these sites was determined.

## Methods

### Patient selection

Inverse potential mapping was performed in ten male patients (mean age 64 ± 5 years old) with an implanted MRI-conditional DDD pacemaker system (Advisa MRI™ Surescan®, Medtronic Inc., Minneapolis, MN, USA) and a structurally normal heart. Patient characteristics are provided in Table 1.

**Table 1:** Patient characteristics

Patient	Age (years)	Pacing indication	PR (ms)	QRS (ms)	QTc (ms)	Relevant comorbidity
1	66	Asystole	138	102	399	Hypertension
2	42	Asystole	148	96	363	Hemochromatosis
3	64	Bradycardia	180	94	384	Hypercholesterolemia
4	69	AV-block	190	180	380	-
5	70	AV-block	216	150	427	-
6	61	SSS + AV-block	314	128	411	Hypertension
7	69	AV-block	204	169	474	–
8	54	Chronotropic Incompetence	204	98	424	Paroxysmal AF
9	65	SSS	268	100	395	–
10	79	Bradycardia	227	126	380	–

*SSS* sick sinus syndrome

The study complied with the declaration of Helsinki and received approval from the local ethical committee and the institutional scientific board. Written informed consent was obtained from all patients.

**Fig. 1:**
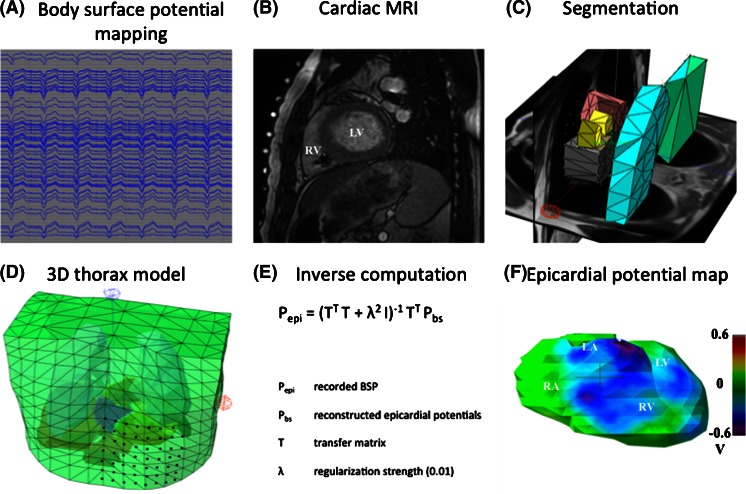
Complete workflow (**a**–**f**) for a non-invasive IPM procedure

An overview of the complete workflow is provided in Fig. 1.

### Body surface potential mapping

Body surface potentials (BSP) were recorded using a 65-channel (62 torso + 3 limb electrodes) ActiveTwo BSPM system with passive electrodes and shielded cables (BioSemi BV, Amsterdam, The Netherlands). The BSP electrodes were positioned on the anterior thorax using a 20-mm inter-electrode distance (Fig. 1d) [7]. BSP were recorded in supine position at a sampling rate of 2048 Hz for 10 s during right ventricular (RV) pacing at a rate exceeding the intrinsic rate with at least 15 beats per minute (paced AV-delay 70 ms).

### Magnetic resonance imaging

After BSPM, MRI markers were applied to replace all torso electrodes. These markers were used to locate the electrode positions on the MRI images, thereby minimizing the systematic error in the inverse procedure. Before entering the MRI room, pacing thresholds, P- and R-wave amplitude, and lead impedance were determined and the pacemaker system was programmed into MRI SureScan® mode [8].

In order to control lung volume, breath holding was practiced with the patient prior to the examination. A stack of ECG-triggered, T2-weighted, bright blood images (slice thickness, 6 mm) was acquired to record the anatomy of the thorax and register the position of the RV lead tip and torso electrodes. Subsequently, cardiac function was assessed using steady state free precession (SSFP) short-axis, three- and four-chamber cines (slice thickness, 6 mm, temporal resolution <50 ms). All images were obtained during breath-hold on a Siemens Aera 1.5 Tesla MRI scanner (Siemens Healthcare, Erlangen, Germany). Cardiac function was analysed using CMR42® software (Circle Cardiovascular Imaging, Calgary, Alberta, Canada).

After the examination, pacing thresholds, P- and R-wave amplitude and lead impedance were determined and compared to the initial values. Finally, original programming of the pacemaker was restored.

### Data processing

#### 3D thorax model

For every patient, a detailed anatomical thorax model, including a 3D whole-heart model, was constructed from the MRI images. Thoracic structures including the heart with all four compartments, lungs, liver and spleen were manually segmented directly in 3D using custom-written software. Conductivities were assigned to each of these structures as known from literature (thorax, 0.2 S/m; lungs, 0.04 S/m; liver, 0.03 S/m and spleen, 0.04 S/m) [9]. A triangulated 3D thorax model was reconstructed using Gmsh software [10].

#### Inverse computation

For each patient, a single paced beat within the 10-s acquisition window was selected for inverse computation. The electrograms do not require any specific editing prior to processing. Epicardial potentials (*P*_epi_) were calculated from the recorded body surface potentials (*P*_bs_) using *P*_epi_ = (*T*^*T*^*T* + *λ*^2^*I*)^−1^*T*^*T*^*P*_bs_ where *T* is the forward transfer matrix and *λ* is the regularization strength (0.01). Finally, an epicardial potential map of a single paced ventricular beat was reconstructed.

#### Ventricular lead tip position and the site of earliest depolarization

An investigator (PB), blinded to the actual ventricular lead tip position, identified the site of earliest depolarization on the colour-coded epicardial potential map. Subsequently, the distance between this site and the position of the ventricular lead tip on the MRI images was determined.

#### Correlation

In order to evaluate the reproducibility of the applied IPM method, two different paced beats were analysed for each patient. The epicardial potential distribution was reconstructed independently. Subsequently, the correlation between the epicardial potentials estimated from the two different beats was calculated using custom-written software.

#### Computing platform

Results are shown as mean ± SD and are expressed as absolute values. All analyses were performed on a 2.4-GHz quad core laptop running the Windows 8 OS. Solving the potential equations was delegated to an Ubuntu 12.10 virtual machine running on this laptop.

## Results

The BSP recording and MRI examination lasted approximately 60 min. None of the patients reported any complaints during or after the MRI examination. Pacing thresholds and leads impedances remained unaffected by the MRI scan in all patients.

**Fig. 2:**
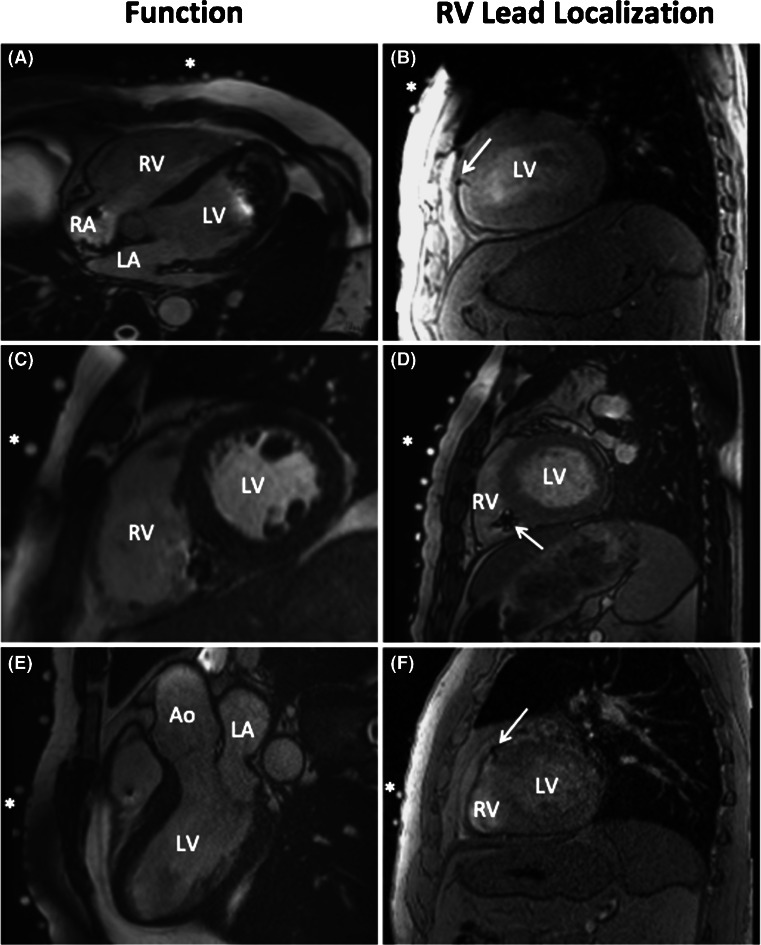
*Left panel*, end-diastolic frames from four-chamber (*A*), short-axis (*C*) and three-chamber (*E*) steady state free precession (SSFP) CINE images. Despite regional artefacts, caused by either the impulse generator or the pacing leads, the image quality allows for accurate assessment of LV and RV volumes and function. *LA* left atrium, *LV* left ventricle, *RA* right atrium, *RV* right ventricle, *Ao* aorta. *Right panel*, three examples (*B*, *D* and *F*) of the T2-weighted, bright blood images used to localize the RV pacing lead (*white arrows*). The *asterisks* indicate the MRI markers that represent the location of the BSP electrodes

Despite the presence of a pacemaker, image quality was good to excellent in all patients and allowed for left and right ventricular function assessment (Fig. 2, panels A, C and E). MRI-derived global function measures are listed in Table 2.

**Table 2:** MRI parameters

Patient	LVEDV (ml)	LVESV (ml)	LVSV (ml)	LVEF (%)	RVEDV (ml)	RVESV (ml)	RVSV (ml)	RVEF (%)
1	147	60	87	59	145	55	91	62
2	133	66	67	50	172	104	69	40
3	135	73	63	46	153	94	58	38
4	193	114	79	41	151	85	66	44
5	153	89	63	41	163	107	55	34
6	175	75	100	57	165	80	86	54
7	187	108	80	43	150	85	66	44
8	157	72	84	54	209	122	87	42
9	177	92	85	48	160	90	70	44
10	113	58	55	48	166	111	55	33

*LVEDV* left ventricular end-diastolic volume, *LVESV* left ventricular end-systolic volume, *LVSV* left ventricular stroke volume, *LVEF* left ventricular ejection fraction, *RVEDV* right ventricular end-diastolic volume, *RVESV* right ventricular end-systolic volume, *RVSV* right ventricular stroke volume, *RVEF* right ventricular ejection fraction

Figure 2 (panels B, D and F) provides typical examples of MRI images used to identify the position of RV lead and the RV lead tip.

### Potential maps

**Fig. 3:**
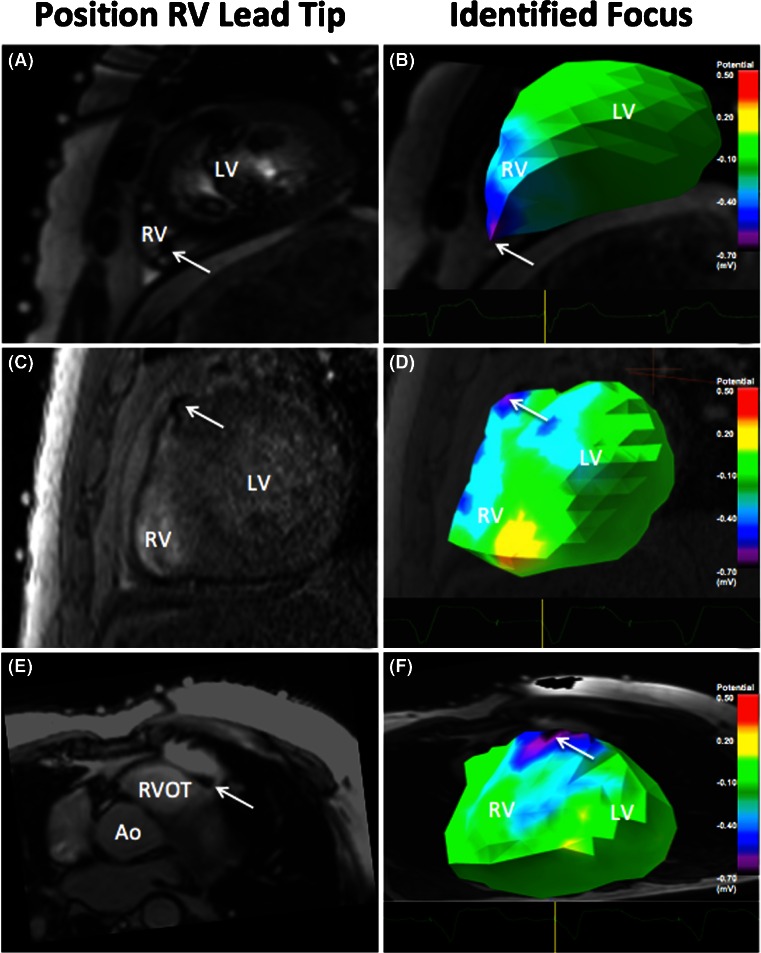
RV lead tip position and site of earliest depolarization on the potential maps. Pacing from the RV apex (*A* and *B*), high in the mid-ventricular septum (*C* and *D*) and right ventricular outflow tract (*E* and *F*)

Epicardial potentials were inversely reconstructed and the distribution was visualized using potential maps. For all patients, the site of earliest ventricular depolarization could be identified. Figure 3 shows three typical examples of the potential distributions reconstructed from the BSP recorded during pacing from different locations.

In two patients (patients 4 and 8), depolarization started in the superior part of the right ventricular septum. A rapid subsequent depolarization of the right ventricular free wall was observed (video 1).

In two patients (patients 1 and 2), the site of earliest depolarization was located in the apical region of the right ventricle. From that point, depolarization spread rapidly to the basal portion of both the RV and left ventricle (LV) (video 2).

### Localization

In patients with the tip of the pacing lead close to the epicardium (apex or right ventricular outflow tract), the distance between lead tip position and epicardial breakthrough was 6.0 ± 1.9 mm.

For the patients with the tip of the pacing lead implanted in the middle of the mid-ventricular septum, the observed site of epicardial breakthrough varied substantially across the right ventricular free wall. In these patients, the intrinsic distance from the lead tip to the epicardium was relatively large (range, 5–30 mm). This distance contributes significantly to the measured localization error (range, 11–45 mm).

### Correlation

**Fig. 4:**
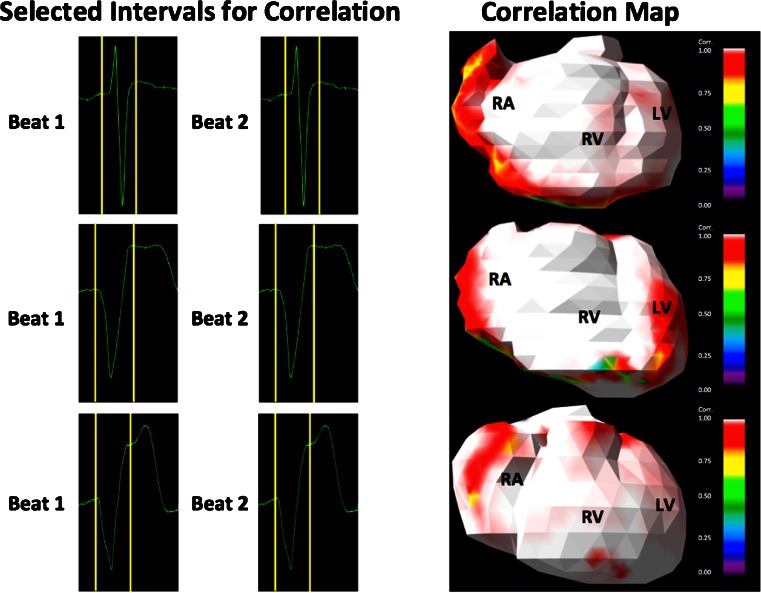
Three examples of intervals selected for correlation analysis (*left panel*) and the corresponding correlation maps (*right panel*). In all patients, a high correlation (*r* = 0.97–0.99, *p* < 0.001) between the epicardial potential distribution of two different paced beats was found

In all patients, a high correlation (*r* = 0.97–0.99, *p* < 0.001) between the epicardial potential distributions of two different paced beats was calculated (Table 3). Correlation values were visualized using correlation maps (Fig. 4).

**Table 3:** Distance epicardial focus to lead tip and correlation between two different paced beats

Patient	Position RV lead tip	Distance epicardial focus to lead tip (mm)	Correlation (*r*)
1	Apex	4	0.996
2	Apex	5	0.997
3	Mid-septum	45	0.978
4	RVOT	6	0.997
5	Mid-septum	20	0.995
6	Infero-septum	25	0.979
7	Mid-septum	11	0.999
8	RVOT	9	0.994
9	Infero-septum	13	0.999
10	Mid-septum	43	0.966

## Discussion

This is one of the first *in vivo* studies in patients with an MRI-conditional pacemaker system, in which a combined MRI and IPM approach was applied to non-invasively determine the origin and epicardial potential distribution during pacing. In patients with the tip of the pacing lead close to the epicardium, a localization error of 6.0 ± 1.9 mm was found.

This clinically relevant accuracy and the less cumbersome nature of the present IPM method may facilitate non-invasive localization of ectopic ventricular foci in patients suffering from ventricular arrhythmia prior to entering the catheterization suite.

In addition, this offers the prospect of thoroughly investigating *in vivo* electrical and mechanical activation patterns in patients with an implanted MRI-conditional pacemaker system.

### Novelty of this work

#### IPM in patients with MRI-conditional pacemaker systems

To our knowledge, this is one of the first reports on the application of IPM in patients with implanted MRI-conditional pacemaker systems. Most validation studies on non-invasive mapping were performed in animal hearts using computed tomography (CT) or in humans during conventional electrophysiological studies [11–14].

In the past, a limited number of patients with an implanted pacemaker have been studied for validation purposes. In these studies, ECGI uses 254 torso electrodes and CT images to calculate epicardial potentials. In only one patient with an implanted cardiac resynchronization therapy (CRT) device, a localization error of 7 mm for the RV pacing lead and 11 mm for the LV lead has been reported [15].

NICE uses 65 electrodes and MRI images to estimate endo- and epicardial activation times. NICE has been applied in ten patients with an implanted CRT device [16] and in one patient with a CRT device and a quadripolar LV lead [17]. No localization errors could be determined in these studies. Due to the non-MRI compatibility of the devices used, the MRI scan was performed prior to implantation of the device, whereas BSP were recorded after implantation. Hence, co-registration of the device, the anatomy of the patients and torso electrodes was not performed.

Recently, Revishili et al. [18] demonstrated the accuracy of a novel non-invasive epi- and endocardial electrophysiology system (NEEES) in 29 patients with implanted devices (26 CRT devices in three dual-chamber pacemakers). The mean distance from the non-invasively predicted pacing site to the anatomic reference site was 10.8 ± 5.4 mm for the right atrium, 7.7 ± 5.8 mm for the right ventricle and 7.9 ± 5.7 mm for the left ventricle. However, the authors state that members processing the NEEES data were not completely blinded for the exact position of each pacing lead on the available CT images.

#### Anterior concentrated electrodes

In this study, all BSP electrodes were positioned in a matrix, directly overlaying the heart. By concentrating the electrodes, a localization error of 6.0 ± 1.9 mm was found for patients with the tip of the pacing lead close to the epicardium. The rapid positioning of this electrode array may facilitate the incorporation of this technique in clinical practice.

#### Epicardial potentials

Currently, only epicardial potentials can be reconstructed from the recorded BSP. Reconstruction of both epicardial and endocardial activation would require the application of an a priori activation model (e.g. NICE, NEEES). Use of a priori models mandates various assumptions to be made and limits the number of pathology related activation patterns that can be represented.

As described previously by Rudy and co-workers, epicardial potentials contain information on intramural and endocardial activation even before the epicardium is activated [19]. Epicardial potentials of the complete cardiac cycle can be non-invasively reconstructed and isochrones or activation times can be derived form the reconstructed potentials [20].

In patients with implanted pacing devices, visualization of epicardial activation may provide additional insight into the efficacy of the pacing strategy. Changes in depolarization or repolarization or other signs of adverse pacing effects may be detected at an early stage. Various studies have demonstrated that long-term ventricular pacing may have various unintended adverse electrophysiological and mechanical effects [21, 22]. In order to better understand the underlying (patho-) physiological mechanisms, it is important to further develop clinically applicable, non-invasive techniques that facilitate an integrated electro-mechanical assessment.

#### MRI

Using MRI as a reference for the RV lead tip position is a novelty. The introduction of MRI-conditional pacemaker systems enables the use of MRI for IPM. Unlike CT, MRI is not associated with radiation exposure. MRI-conditional devices can be programmed in an MRI safe mode, which makes the device less susceptible to the magnetic energy of the MRI environment and decreases the risk of hard- or software interactions.

Besides visualizing the lead position, MRI may provide valuable information on the mechanical activation in patients with implanted pacemaker systems. In contrast to CT, MRI enables the safe performance of follow-up studies. In addition, MRI is the gold standard for the detailed assessment of atrial and ventricular volumes, function and wall motion abnormalities [23]. Moreover, the presence and the extent of myocardial scar tissue can be determined, which may influence cardiac electrical activation patterns and may impact the effects of pacing [24].

The presence of an implanted device generates image artefacts that may compromise image quality. By adapting the scan strategy, the amount of artefacts produced by the implanted pacemaker system can be kept at a minimum [25]. Ventricular function is usually assessed in the short-axis view. These images are mildly affected by the distortion caused by the presence of a pacemaker. Left and right ventricular function could be assessed for all patients in this study.

### Limitations of the study

The site of epicardial breakthrough on the epicardial potential map was compared to the position of the ventricular lead tip on MRI and the distance between these points was determined. The position of the RV lead tip was determined using the susceptibility artefact caused by the lead tip on the MRI images (Figs. 2 and 3). Alternatively, CT may be used for this purpose. Nevertheless, the scattering of radiation causes the overall image quality to decline. In addition, the metallic artefact caused by the lead will hamper the localization of the tip of lead [26].

For several patients, the reported distance from the lead tip to the site of earliest depolarization on the epicardial potential maps includes the distance from the lead tip in the septum to the epicardium. Septal insertion of the lead tip increases the intrinsic distance from the lead tip to the site of epicardial breakthrough. This is a limitation of the reconstruction of epicardial potentials. Therefore, the localization accuracy is probably severely underestimated in this group of patients.

The anterior concentration of BSP electrodes does not allow for accurate investigation of depolarization on the posterior wall. This pilot study focussed on investigating the feasibility of performing IPM in patients with an implanted MRI-conditional pacemaker system. The current research did not describe the accuracy of localizing foci in the inferior wall of the RV or LV. Atrial stimuli were not analysed in the current study.

It has been shown that prematurity of ectopic foci can affect cardiac activation patterns. This was not explored in this current study by introducing different coupled extra-stimuli during RV pacing.

The accuracy of localizing epicardial breakthrough may be affected by extensive scar in patients with prior infarction or severe cardiomyopathy. For this reason and in consideration of the limited size of the study population, additional research needs to be performed to further explore the clinical benefit of IPM in combination with MRI in patients suffering from a wide range of pathologies.

## Conclusion

In this study, an IPM method using a limited number of electrodes was applied in patients with an MRI-conditional pacemaker system. For all patients, the epicardial potential distribution could be reconstructed from BSP recorded during pacing. In patients with the tip of the pacing lead close to the epicardium, a localization accuracy of 6.0 ± 1.9 mm was found. Application of this method in patients suffering from ventricular arrhythmia may enable accurate non-invasive localization of ectopic ventricular foci prior to entering the catheterization suite.

## Electronic supplementary material

Video 1 This video shows a typical example of a patient with the RV lead implanted in the RVOT. First, the lead tip is identified on the MRI images. Second, the epicardial potentials map is shown. This map is reconstructed from body surface potentials recorded during ventricular pacing. The area of earliest depolarization corresponds to the region of the lead tip. Subsequently, a rapid depolarization of the right ventricular free wall is observed. (MP4 1.91 mb)

Video 2 This video shows a typical example of a patient with the RV lead implanted in the RV apex. After identification of the lead tip, the epicardial potentials map is shown. The area of earliest depolarization corresponds to the region of the lead tip. From this point, depolarization spreads rapidly to the basal portion of both the RV and LV. (MP4 1.42 mb)

## Abbreviations

BSP: Body surface potentials
BSPM: Body surface potential mapping
CRT: Cardiac resynchronization therapy
CT: Computed tomography
ECG: Electrocardiogram
ECGI: Electrocardiographic imaging
EP: Electrophysiological
IPM: Inverse potential mapping
LV: Left ventricle
MRI: Magnetic resonance imaging
NICE: Non-invasive imaging of cardiac electrophysiology
RV: Right ventricle
RVOT: Right ventricular outflow tract
SSFP: Steady state free precession

## References

[1] Erkapic D, Greiss H, Pajitnev D, Zaltsberg S, Deubner N, Berkowitsch A (2015). Clinical impact of a novel three-dimensional electrocardiographic imaging for non-invasive mapping of ventricular arrhythmias—a prospective randomized trial. Europace.

[2] Jamil-Copley S, Bokan R, Kojodjojo P, Qureshi N, Koa-Wing M, Hayat S (2014). Noninvasive electrocardiographic mapping to guide ablation of outflow tract ventricular arrhythmias. Heart Rhythm.

[3] Vijayakumar R, Silva JN, Desouza KA, Abraham RL, Strom M, Sacher F (2014). Electrophysiologic substrate in congenital long QT syndrome: noninvasive mapping with electrocardiographic imaging (ECGI). Circulation.

[4] Berger T, Fischer G, Pfeifer B, Modre R, Hanser F, Trieb T (2006). Single-beat noninvasive imaging of cardiac electrophysiology of ventricular pre-excitation. Journal of the American College of Cardiology.

[5] Bokeriia LA, Revishvili AS, Kalinin AV, Kalinin VV, Liadzhina OA, Fetisova EA (2008). Hardware-software system for noninvasive electrocardiographic examination of heart based on inverse problem of electrocardiography. Meditsinskaia Tekhnika.

[6] Ferreira AM, Costa F, Tralhão A, Marques H, Cardim N, Adragão P (2014). MRI-conditional pacemakers: current perspectives. Medical Devices (Auckl).

[7] van der Graaf, A. W., Bhagirath, P., de Hooge, J., de Groot, N. M., Götte, M. J. (2015) A-priori model independent inverse potential mapping; the impact of electrode positioning. *Clinical Research in Cardiology*. In press. 10.1007/s00392-015-0891-7 PMC4712232 26216293

[8] Wollmann CG, Thudt K, Kaiser B, Salomonowitz E, Mayr H, Globits S (2014). Safe performance of magnetic resonance of the heart in patients with magnetic resonance conditional pacemaker systems: the safety issue of the ESTIMATE study. Journal of Cardiovascular Magnetic Resonance.

[9] Oostendorp T, Nenonen J, Korhonen P (2002). Noninvasive determination of the activation sequence of the heart: application to patients with previous myocardial infarctions. Journal of Electrocardiology.

[10] Marchandise E, Geuzaine C, Remacle JF (2013). Cardiovascular and lung mesh generation based on centerlines. International Journal of Numerical Methods in Biomedical Engineering.

[11] Oster HS, Taccardi B, Lux RL, Ershler PR, Rudy Y (1998). Electrocardiographic imaging: noninvasive characterization of intramural myocardial activation from inverse-reconstructed epicardial potentials and electrograms. Circulation.

[12] Cakulev I, Sahadevan J, Arruda M, Goldstein RN, Hong M, Intini A (2013). Confirmation of novel noninvasive high density electrocardiographic mapping with electrophysiology study: implications for therapy. Circulation. Arrhythmia and Electrophysiology.

[13] Sapp JL, Dawoud F, Clements JC, Horacek BM (2012). Inverse solution mapping of epicardial potentials: quantitative comparison with epicardial contact mapping. Circulation. Arrhythmia and Electrophysiology.

[14] Ghanem RN, Jia P, Ramanathan C, Ryu K, Markowitz A, Rudy Y (2005). Noninvasive electrocardiographic imaging (ECGI): comparison to intraoperative mapping in patients. Heart Rhythm.

[15] Ramanathan C, Ghanem RN, Jia P, Ryu K, Rudy Y (2004). Electrocardiographic imaging (ECGI): a noninvasive imaging modality for cardiac electrophysiology and arrhythmia. Nature Medicine.

[16] Berger T, Pfeifer B, Hanser FF, Hintringer F, Fischer F, Netzer M (2011). Single-beat noninvasive imaging of ventricular endocardial and epicardial activation in patients undergoing CRT. PloS One.

[17] Seger M, Hanser F, Dichtl W, Stuehlinger M, Hintringer F, Trieb T (2014). Non-invasive imaging of cardiac electrophysiology in a cardiac resynchronization therapy defibrillator patient with a quadripolar left ventricular lead. Europace.

[18] Revishvili, A. S., Wissner, E., Lebedev, D. S., Lemes, C., Deiss, S., Metzner, A. et al. (2015). Validation of the mapping accuracy of a novel non-invasive epicardial and endocardial electrophysiology system. *Europace.* Feb 2. 10.1093/europace/euu339 PMC4535554 25643987

[19] Rudy Y (2013). Noninvasive electrocardiographic imaging of arrhythmogenic substrates in humans. Circulation Research.

[20] Oster HS, Taccardi B, Lux RL, Ershler PR, Rudy Y (1997). Noninvasive electrocardiographic imaging: reconstruction of epicardial potentials, electrograms, and isochrones and localization of single and multiple electrocardiac events. Circulation.

[21] De Sisti A, Márquez MF, Tonet J, Bonny A, Frank R, Hidden-Lucet F (2012). Adverse effects of long-term right ventricular apical pacing and identification of patients at risk of atrial fibrillation and heart failure. Pacing and Clinical Electrophysiology.

[22] Akerström F, Pachón M, Puchol A, Jiménez-López J, Segovia D, Rodríguez-Padial L (2014). Chronic right ventricular apical pacing: adverse effects and current therapeutic strategies to minimize them. International Journal of Cardiology.

[23] Abbasi SA, Ertel A, Shah RV, Dandekar V, Chung J, Bhat G (2013). Impact of cardiovascular magnetic resonance on management and clinical decision-making in heart failure patients. Journal of Cardiovascular Magnetic Resonance.

[24] Bleeker GB, Kaandorp TA, Lamb HJ, Boersma E, Steendijk P, de Roos A (2006). Effect of posterolateral scar tissue on clinical and echocardiographic improvement after cardiac resynchronization therapy. Circulation.

[25] Khan JN, Singh A, Pakkal MV, McCann GP (2013). MRI-safe pacemakers and reduction of cardiac MRI artefacts with right-sided implantation. European Heart Journal and Cardiovascular Imaging.

[26] Sasaki T, Hansford R, Zviman MM, Kolandaivelu A, Bluemke DA, Berger RD (2011). Quantitative assessment of artifacts on cardiac magnetic resonance imaging of patients with pacemakers and implantable cardioverter-defibrillators. Circulation Cardiovascular Imaging.

